# First-time mothers’ experiences of pregnancy and birth following assisted reproductive technology treatment in Taiwan

**DOI:** 10.1186/s41043-019-0167-3

**Published:** 2019-03-29

**Authors:** Mei-Zen Huang, Yi-Chin Sun, Meei-Ling Gau, Shuby Puthussery, Chien-Huei Kao

**Affiliations:** 1 Department of Nursing, National Tainan Junior College of Nursing, 78, Sec.2 Minzu Rd, Tainan City, Taiwan; 2 Department of Nursing, National Taipei University of Nursing and Health Sciences, 365, Ming-Te Road, Peitou, Taipei, Taiwan; 3 Department of Midwifery and Women Health Care, National Taipei University of Nursing and Health Sciences, 365, Ming-Te Road, Peitou, Taipei, Taiwan; 4 Maternal and Child Health Research Centre, Institute for Health Research & School of Health Care Practice, University of Bedfordshire, Putteridge Bury, Luton, Bedfordshire, LU2 8LE UK

**Keywords:** Assisted reproductive technology, Infertility, Mothers, Qualitative study

## Abstract

**Background:**

Assisted reproductive technology (ART) treatment tends to involve significant physical and emotional commitments that can impact maternal, infant, and family health and well-being. An in-depth understanding of experiences is necessary to provide adequate support for women and their families during pregnancy and transition to parenthood following ART treatment. The aim of this study was to explore first-time mothers’ experiences of pregnancy and transition to parenthood following successful ART treatment in Taiwan.

**Method:**

Twelve first-time mothers who conceived and gave live birth using ART treatment were purposively selected from a fertility centre in Taipei, Taiwan. Women’s experiences in pregnancy and in their transition to motherhood were explored using semi-structured in-depth interviews. All interviews were recorded, transcribed, and analysed using the Colaizzi strategy.

**Results:**

The mothers’ accounts reflected three main themes: ‘being different from mothers who became pregnant naturally’, ‘ensuring health and safety of the foetus’, and ‘welcoming new lives with excitement’. The difference mothers felt about themselves was evident in four subthemes: becoming pregnant after a long wait, feeling vulnerable during pregnancy, relying on family’s assistance and support, and worrying about the impact of ART on health. The theme on ‘ensuring health and safety of the foetus’ encompassed three subthemes: activities to protect the unborn baby, monitoring foetal movement constantly to maintain peace of mind, and receiving foetal reduction for the sake of the pregnancy. Narratives around ‘welcoming new lives with excitement’ reflected four subthemes: overcoming hardship for worthwhile results, realising one’s life and dreams, proving to be fertile enough to give birth, and return to normal life track.

**Conclusion:**

Findings indicate the need for educational and psychosocial interventions to support women and their families physically and psychologically during ART treatment. The stigma related to infertility and the psychosocial support from family are aspects to consider while planning intervention programmes.

## Background

Assisted reproductive technology (ART) has become a widely accepted method to enable women with untreatable infertility to conceive healthy babies. Estimates suggest that more than seven million babies were born worldwide within the last four decades as a result of successful ART since the first baby was born via ART in 1978 [1]. While this method of reproduction allows women to circumvent infertility, it may not always produce the desired outcomes [2]. The perceptions of conception, pregnancy, and motherhood may change for women who conceive through ART, and the pregnancy may carry a different beginning for them [3]. ART treatment tends to involve significant physical and emotional commitments on the part of the couple, and some researchers have linked conception through ART to issues such as delayed mother-infant attachment, reduced maternal confidence, low self-esteem, increased anxiety about pregnancy loss, and overprotective parenting [4–7]. Hammarberg et al. [7] in their systematic review found higher anxiety about the survival of the foetus and early parenting difficulties and lower post-natal self-confidence among ART couples compared to others. The authors, however, noted that the evidence about adjustment to pregnancy, parenthood, and parental perceptions of infant temperament and behaviour was inconclusive [7]. It has been argued that infertility treatment may be associated with levels of distress over and above those associated with the state of being infertile in and of itself [8]. The burden of infertility and the inability to conceive naturally may remain for women even after conception through ART [9]. First-time mothers, after successful ART treatment, may be affected by high anxiety levels and concerns about their child’s health, often resulting in over-protective parenting behaviour [10].

Some studies have reported lower or similar levels of anxiety and depression among women who conceived via ART compared to those conceiving naturally [11–14]. In their systematic review of the psychological health of women undertaking ART, Verhaak et al. [14] found that pregnancy via successful ART treatment helped to reduce the mothers’ anxiety, depression, and stress. As a coping mechanism, women who had prior failed attempts at ART often viewed their pregnancies as special or considered the procedures normal, which enabled them to cope with the demands [15]. The sense of fulfilment experienced by mothers who had become pregnant via ART tend to override any uncertainties about their loss of independence and the financial impact of having a child [12].

Cultural variations in perspectives relating to pregnancy, childbirth, and parenting may also influence women’s experiences of pregnancy and birth after ART. A study from Brazil reported that the pregnancy experience itself after ART was regarded by the women as a reward or compensation for the difficulties, although undergoing the treatment might be emotionally painful, particularly if previous treatments have failed [3]. The authors found differing perspectives depending on whether the pregnancy followed the first ART. Those who have had previously unsuccessful treatments were less concerned about the process but were more concerned about possible physical problems [3]. Another study found the perspectives of couples in Italy were centred around the temporal dimension and represented the contradictory emotions and expectations that they experienced during the treatment journey [16].

Although qualitative studies are emerging globally as described above, in-depth evidence is scarce concerning women’s experiences of successful ART treatment and ensuing pregnancy, childbirth, and parenting. According to the Bureau of Health Promotion in Taiwan, approximately 20% of infertile women undergo IVF treatment in which 36% of these women become pregnant. Approximately 1.8% of babies born in Taiwan are conceived via ART [17]. Women over 35 years of age account for 67.5% of those who undergo IVF treatment [18].

The only existing qualitative study from Taiwan by Lin et al. [19] on Taiwanese women’s experiences of pregnancy was conducted 1 year after the baby’s birth and offered some relevant insights. The participants in this study had undergone at least three cycles of ART over a period of more than 3 years. The authors reported that while the safety and health of the foetus was the primary concern for the mothers, other concerns such as physical/physiological changes, psychosocial reactions, the transition of identity during pregnancy, and the impact of Taiwanese society on the pregnancy were also prevalent among the mothers [19]. The current study was conceived to provide additional in-depth evidence required to develop appropriate interventions to women and their families undergoing ART treatment in Taiwan and other settings internationally. The aim of the study was to understand first-time mothers’ experiences of pregnancy and transition to parenthood following successful ART treatment in Taiwan.

## Methods

### Design

The data presented in this paper are derived from an in-depth qualitative study based on a phenomenological approach conducted at a fertility centre in Taiwan. The team included experienced researchers from various backgrounds such as nursing, midwifery, public health, and qualitative research. All the researchers were females.

### Recruitment and participants

The participants were 12 pregnant women who conceived and gave live birth using ART at the fertility centre in Shin Kong Wu Ho-Su Memorial Hospital, Taipei, Taiwan. The inclusion criteria for participation were that the women gave live birth to the first baby following ART treatment; they did not have a history of medical complications such as high blood pressure, diabetes, and heart disease before they got pregnant; and they could understand, speak, read, and write Mandarin. In order to include a range of experiences and to enhance the transferability of the research findings, women who had given birth to singletons or twin babies as well as those who had premature or full-term births were included.

After gaining relevant ethical approval from the ethics committee in Taiwan, the principal investigator (MzH) approached potential participants at the fertility clinic at Shin Kong Wu Ho-Su Memorial Hospital, Taipei, Taiwan, with an information leaflet. Those who were interested in participating after the initial discussion were given detailed information about the study and what participation involved. Written consent was obtained before they were enrolled in the study.

The characteristics of the participants are presented in Table 1. Their ages ranged from 31 to 36 years. All were married. Most of the women had university level education with bachelor’s or master’s degrees. The number of foetuses conceived following ART ranged from 1 to 4, with seven women conceiving three or more foetuses and subsequently undergoing foetal reduction. Most of the pregnancies (*n* = 8) resulted in twins. Two women reported pregnancy complications, with one reporting placenta accrete (*n* = 1) and the other gestational diabetes mellitus (GDM).

**Table 1:** Participant characteristics

Participant	Age	Occupation	Married	Education	Number of foetus	Foetus reduction	Number of live babies	Complications during pregnancy	Type of complication	Postpartum week interviewed
A	31	None	Yes	Bachelor’s degree	3	1	2	No	–	13
B	33	Hospital administrator	Yes	Bachelor’s degree	3	1	2	Yes	Placenta accreta	9
C	35	None	Yes	Master’s degree	4	2	2	No	–	18
D	39	None	Yes	Bachelor’s degree e	3	1	2	No	–	10
E	35	None	Yes	Bachelor’s degree	3	1	2	Yes	Gestational diabetes mellitus	8
F	36	None	Yes	Bachelor’s degree	4	2	2	No	–	11
G	30	Books service	Yes	Master’s degree	2	–	2	No	–	8
H	36	None	Yes	Associate degree	1	–	1	No	–	13
I	37	None	Yes	Bachelor’s degree	1	–	1	No	–	14
J	37	Hospital administrator	Yes	Associate degree	1	–	1	No	–	14
K	36	None	Yes	Associate degree	1	–	1	No	–	8
L	36	Bank administrator	Yes	Bachelor’s degree	3	1	2	No	–	12

### Data collection

The in-depth interviews took place 8–18 weeks after the women gave birth. All of the interviews were conducted by the first author (MzH) who has 7 years of nursing experience in the delivery room, postpartum ward, and paediatric care unit of the hospital. The interviews were conducted at the participants’ homes at a mutually convenient prearranged time, and there was no one else present apart from the researcher and the participant during the interview. A flexible topic guide was used for the interviews. The women were first asked in general about their pregnancy and childbirth and then about the experiences of and feelings towards the ART treatment process and their subsequent motherhood. The topic guide included questions on experiences during treatment; decisions about foetal reduction if needed; reactions from the extended family; how they coped with family chores and employment; views about baby’s health and well-being; and expectations about additional support required from health care professionals before and during pregnancy, at childbirth, and after the delivery.

In order to ensure the credibility of the data, probing questions such as ‘You just mentioned… would it be possible to talk a little more about it please?’ were used. The interviews lasted between 1 and 2 h, guided by the woman’s desire to talk. The interviewees were given time to think about and reflect upon their experiences before the interviews and were assured that anything they said would be valued and respected. The interviews were audio-recorded with permission from the participants. The researcher also took notes during the interviews. The final sample size was determined based on data saturation where no new information was forthcoming.

### Analytical approach

All of the interviews were transcribed. Each transcript was about 30 pages long on average and contained very rich data. After the preliminary analysis of the transcripts, the researcher invited the interviewees to confirm the transcript contents in order to assure the credibility of the data. The texts were then coded and analysed based on Colaizzi’s method [20]. We adopted Colaizzi’s method as it offered a clear and systematic approach to provide a concise, yet comprehensive description of the phenomenon under study along with opportunities for participant validation [21]. The first stage of the analysis involved close reading of the interview transcripts several times and extracting significant statements and phrases. The significant statements were coded and the initial codes were then grouped into more abstract levels of codes or themes. The analysis was done manually. The first author took the lead in coding the data, and the codes were regularly reviewed by the other authors to check for accuracy and consistency.

## Results

The interviews reflected three key themes as presented in Table 2: being different from mothers who became pregnant naturally, ensuring the health and safety of the foetus, and welcoming new lives with excitement.

**Table 2:** Key themes and sub-themes

Theme	Sub-theme
1. Being different from mothers who became pregnant naturally	1) Becoming pregnant after a long wait
	2) Feeling vulnerable during pregnancy
	3) Relying on family’s assistance and support
	4) Worrying about the impact of ART on health
2. Ensuring health and safety of the foetus	1) Activities to protect the unborn baby
	2) Monitoring foetal movement constantly to maintain peace of mind
	3) Receiving foetal reduction for the sake of the pregnancy (presented in detail elsewhere)
3. Welcoming new lives with excitement	1) Overcoming hardship for worthwhile results
	2) Realising one’s life and dreams
	3) Proving to be fertile enough to give birth
	4) Return to normal life track

### Being different from mothers who became pregnant naturally

Generally, the mothers reflected a feeling of being different from mothers who conceived naturally. The difference they felt about themselves was evident in four subthemes: becoming pregnant after a long wait, feeling vulnerable during pregnancy, relying on family’s assistance and support, and worrying about the impact of ART on health.

#### Becoming pregnant after a long wait

The average time that the participants spent trying to become pregnant was 3.5 years. After such a long wait, becoming pregnant via ART gave the women a feeling of excitement and joy:

When finding out about the pregnancy, I felt really happy. It was a long time since knowing that I could not become pregnant naturally, to have the successful test tube result. (Participant A)

I was thrilled to find out about the pregnancy, it felt like my life turned from black and white to colours. It always feels something missing about the infertility because a child is an integral part of a family. Both my husband and I myself love children, so we both were really happy after knowing about the pregnancy. (Participant G)

Although the mothers expressed feelings of excitement having conceived, they were also gripped with feelings of uncertainty about the outcome of their pregnancy. For those with a history of miscarriage, the fear of losing the pregnancy was even worse:

I felt scared after pregnancy, because the last pregnancy resulted in abortion after 5 weeks. I have to admit that I was too careful about this time, worried that I might lose it again, and did not get out of the door to continue the previous life until 3 months’ pregnant. (Participant I)

I was quite happy about it [the pregnancy]. But I was also happy last time and [the pregnancy] ended up with abortion. I was very disappointed afterward. So I kept telling myself not to be too happy this time, just in case it is lost again. (Participant H)

At the beginning of pregnancy, many women kept the news to themselves due to concerns about the chances of miscarriage. They also feared the potential reactions from family members and friends in the event of losing the baby and therefore waited to disclose the pregnancy:

I didn’t tell colleagues until it was nearly three months, because I was worried that the foetus might disappear. (Participant A)

I was very happy about the first pregnancy and told everyone. Then I lost it. I felt terrible about it and even more so when facing people. So we decided not to tell others about the pregnancy this time. (Participant J)

#### Feeling vulnerable during pregnancy

Although the women felt that pregnancy had overcome their infertility, they felt very vulnerable during their pregnancy. As the pregnancy progressed, the women became increasingly worried about various examination results, the health and development of the foetus, and the complications during pregnancy. Many women described their pregnancy as a series of challenges, one after another. In general, the women felt that becoming pregnant was ‘not a simple happy ending’ in itself:

After the traditional unstable period passed, we were told that we still needed to be careful…even after the fourth and seventh months, and that felt quite uneasy. (Participant H)

The whole pregnancy process was full of worries every week, during every examination, that something might be abnormal or wrong. So becoming pregnant is not a simple happy ending. (Participant E)

The unpredictable future of the pregnancy itself put these women at unease until the baby was born safely:

I was worried through the entire pregnancy until the baby was born safely. (Participant F)

The participants felt that they were constantly under psychological pressure to make the pregnancy successful and took every precaution to make it a success:

The biggest challenge was the psychological pressure … You knew he was there, but that did not mean he would continue to be there in the future, and no one could guarantee that. So I couldn’t help being worried all the time. (Participant E)

After so many efforts and the previous disappointment over pregnancy, I was very cautious this time and worried that I might lose the baby if careless. My heart was up in the air, not knowing if it would be successful or not, I finally became pregnant and it’s better to listen to the advice, hence decided to rest in bed for three months, bearing the discomfort and hoping that the baby then would stay in me safe and sound. (Participant H)

The women felt that the psychological pressure mounted when they sensed any changes in the foetus, although some of these changes would have been normal. For example, less frequent or less obvious foetal movement and early contractions caused intense worry to some of the women:

I knew someone whose baby died in her. That my foetal movement was not very frequent made me very nervous and worried about losing it. It was worrying not to be able to feel the foetal movement. As it became bigger, I felt perhaps the baby was not being active, and concerned something might happen. (Participant D)

Since the twentieth gestational week, I started to feel contractions and tried to lie in bed as much as possible. I was worried... (Participant F)

The complications during pregnancy worsened their fears of unsuccessful pregnancy outcomes. Four of the participants experienced vaginal bleeding at the early stage of pregnancy, which made them feel extremely vulnerable:

I had bleeding at early pregnancy and was worried about losing the baby. Since mid-gestation, I started to worry about the possible premature delivery. (Participant I)

Various medical examinations that the women underwent also appeared to worsen their fears of pregnancy failure. Some of the older women refused to undergo invasive investigations and opted for less invasive tests, although they perceived that the results may be less accurate:

Despite my maternal age, I chose not to do the amniocentesis because I was scared that might harm him and the probability of three-thousandths might happen to me…Testing maternal blood reveals the health condition of three kinds of chromosomes only. If there was any problem with other chromosomes, as long as it doesn’t threaten the baby’s life, I would still choose to keep him. (Participant K)

Many of the women were worried about potential adverse outcomes for the baby, including chromosomal anomalies and preterm birth. As the participants were relatively older women, they perceived a high risk of conditions related to chromosomal anomalies:

I was worried about Down syndrome because of my age. It was torturing while waiting for the result of chorionic villus sampling, because you had no idea if the baby was healthy or not. (Participant D)

I consider my maternal age being old. When I was due for the amniotic fluid test, I was hesitating whether to do it or not because there was a worry about the baby’s condition. My worry lasted until the amniocentesis result came out as normal. (Participant I)

Premature delivery was another source of constant worry:

I was worried about premature delivery hence kept telling families to pay attention to me. I was very nervous about the premature delivery and therefore very cautious about everything at home. (Participant B)

Seven participants continued their pregnancy with twin foetuses, sometimes doing so against medical advice for foetal reduction and the possibility of a higher risk of preterm delivery:

Finally we decided to have twins, and the whole process was really worrying. There is a bigger chance to have premature delivery with twins and with the specialist’s advice (to have a single foetus), which was based on my height and health condition, I still wanted to take the risk so had to be more careful than usual. (Participant F)

#### Relying on family’s assistance and support

From the women’s accounts, it was clear that after years of infertility, the pregnancy was treated as a huge family event. In order for the baby to be delivered safely, all of the family members, including parents, parents-in-law, sisters, and sisters-in-law, supported the mother-to-be and this support worked well for the women in looking after themselves. The women’s accounts showed the intense support that they received from family members:

The whole family made a lot of efforts to make the pregnancy successful. My families cooked for me through the entire pregnancy process. I suspended my job after 5 months’ pregnancy and my mother came to look after me since then. (Participant B)

My parents wanted me to give birth in the United States for the better future of the baby, and they paid for it. My husband couldn’t leave due to his work, so two of my sisters went to America with me to keep me company until the delivery. (Participant C)

In order to support the women through the pregnancy process safely, their families usually took over the housework:

Whatever I wanted to do during pregnancy, families tried their best to support me. I suspended work and rested in bed, my sister who lived nearby was doing my meals every day. My parents-in-law wanted me to give birth in America so they could take care of me and the baby. Two weeks before the expected caesarean, my husband took annual leave and came to America to look after me as well. (Participant H)

#### Worrying about the impact of ART on health

The women were worried about the safety of ART both in terms of its impact on their health as well as that of the baby. From various channels such as books, magazines, media, and internet, the women learned that ART could have potential adverse effects, including an increased risk of cancer, which made them often worry about jeopardising their own health in the long term.

It might not feel anything short term with the ovulation injection, but I believe in the long term or after many injections, it will affect my health. (Participant F)

I read on the Internet that using inducing medicine would increase the mother’s risk of getting cancer. (Participant A)

When reading about the probability of me having cancer is higher than the average, I was very worried. At the beginning, I felt wrong because test tube pregnancy is not natural and it would affect my health. When pregnant, I was worried about how to look after two children if there was any problem with my health and cancer. (Participant B)

They also recounted the worries from family members about the impact on their health:

A family friend had breast cancer or ovarian cancer and was going to have an operation. She blamed the inducing injection she had for the test tube. So my families became very worried about me and kept asking me to be careful. (Participant H).

There were differing opinions about the impact on their babies’ health. Some women thought their babies might have problems such as genetic insufficiency, slow development, or hyperactivity:

I read some articles online about those test tube babies; it seems that they would have some genetic problems. (Participant A)

Because it was not produced in natural course, I suspect there would be many issues such as slow development and unhealthy parts, but it was too early to judge. (Participant E)

I heard that test tube babies are usually overactive, but I couldn’t tell now, perhaps I will know more in the future. (Participant F)

Others believed that as ART facilitates babies to be carefully chosen clinically, they should be healthier than those born naturally:

Test tube selects the best. The clinical team helped to find the best sperm and the ovaries to match; it is unlikely that there will be something wrong with the baby. We feel the test tube baby will be healthy, otherwise how come it has been chosen in the first place? If it is not healthy, how could the sperm and the ovaries marry each other and continue to grow? (Participant J)

### Ensuring the health and safety of the foetus

This theme addresses the accounts about the change in lifestyle behaviours and precautions that the women undertook to ensure the well-being of the foetus. There were three subthemes: activities to protect the unborn baby, monitoring foetal movement constantly to maintain peace of mind, and receiving foetal reduction for the sake of the pregnancy.

#### Activities to protect the unborn baby

To ensure the unborn child’s safety, most of the employed women quit their jobs at some stage: two at the beginning of the treatment, one after the implantation, and one after the first trimester:

After spending so much time and money, we couldn’t stand any risk. So after five month’s pregnancy, I chose to give up working in order to ensure the baby in me is absolutely well taken care of. (Participant A)

In general, the women reduced physical activity as a whole, with many staying indoors at home during pregnancy:

From other mothers of twins I learned that those having twins should not take too much exercise. Previously I liked jogging, but now I would try to avoid heavy activities and just do the minimum. I tried to rest as much as possible at home, and did not really carry any heavy stuff. (Participant G)

I hardly went out, even when I did, I tried not to walk about too much, and wouldn’t exhaust myself. I would try to keep my energy because I wasn’t sure whether I could physically deal with the unexpected circumstances. I was quite reserved about leaving the house and tried to rest as much as possible. But I did not lie in bed all the time. (Participant F)

In addition to reducing their daily activities, the women took various actions to benefit healthy foetal development, such as adhering to a diet that they believed was nutritious:

Nutrition must be provided sufficiently. I had chicken extract every day for the baby to have enough nutrition. (Participant G)

The women also tried to keep themselves happy during pregnancy, which they thought was important to help foetal development:

I promised myself that I’d take laid-back attitude to life and to the baby, trying to stay in a happy mood. (Participant F)

I kept myself happy, which I believe is good for the baby. (Participant G)

#### Monitoring foetal movement constantly to maintain peace of mind

During pregnancy, the women monitored foetal movement constantly to ensure the foetus’ safety and to assure themselves. Any reduction they felt in foetal movement was a source of immense worry:

I didn’t have a lot of foetal movement, which made me very nervous. I was keeping a close eye on foetal movement on daily basis. (Participant J)

As they grew, sometimes I would feel the baby was not particularly active and that worried me. I always paid attention to the foetal movement. (Participant D)

Some women purchased a foetal heart monitor, which enabled them to listen to the foetal heartbeat and confirm that the baby was fine:

I even bought a device to listen to the foetal heartbeat, and listed to it three times a day. When I had a big meal, the baby remained still and I couldn’t help becoming worried. Therefore, I often went to the hospital for examinations, which enabled me to know that the baby was fine, that put my heart at ease. (Participant I)

#### Receiving foetal reduction for the sake of the overall pregnancy outcome

Receiving foetal reduction for the sake of the overall pregnancy outcome was a recurrent theme that came up in the interviews. This is being published in detail in another paper.

### Welcoming new lives with excitement

This theme reflects how the women endured the painful process of their pregnancy and their feelings of fulfilment after giving birth. The narratives reflected four subthemes: overcoming hardship for worthwhile results, realising one’s life and dreams, proving to be fertile enough to give birth, and return to a normal life track.

#### Overcoming hardship for worthwhile results

A successful end to the pregnancy and the start of a new life as parents made the women feel that the physical and psychological hardships they endured were worthwhile:

Treating the infertility is a very hard process. Once successfully giving birth, I felt all of the hard work was worthwhile. (Participant F)

I have a baby now and feel all of the hard work was worthwhile. That I can finally have my own child helps me forget about how I managed to go through all of it, including the physical and psychological struggling and pain. Money and time devoted on this was nothing. I didn’t even know how I managed to go through all of this. When the baby was born, I was so happy. All of the hard work was nothing, and I could certainly forget about the challenging process. (Participant A)

#### Realising one’s life and dreams

The arrival of the baby made the women feel that they had finally realised their dreams of a complete and cohesive family. The women also felt that as a couple, they became more responsible and paid more attention to their own health and well-being in order to be able to bring up the baby. They perceived that motherhood was a realisation of their dreams of pursuing a complete life and made them feel that this resulted in a more motivated, busier, and happier life:

Children make the family complete. (Participants C and J)

After having a baby, the family became complete and more motivated to make things happen. Previously we did not plan to have babies and being just two of us; we felt some fun was missing in life. Suddenly the babies are here and we become very hopeful and do more to keep healthy because I have to keep healthy to look after them. (Participant B)

Children make the family close to each other. My husband takes his responsibility seriously and would like to help the children’s development. (Participant G)

Life becomes so enriched with a baby. (Participant E)

#### Proving to be fertile enough to give birth

The birth of a child was also an opportunity for the women to prove their fertility to their family and friends:

In festive seasons, families and friends always asked why I did not have children. It really was a lot of pressure to say that I could not get pregnant naturally. Now that I have given birth, I can finally have my own children. (Participant A)

The fact that we are infertile made us quite suffer. Finally we managed to use our own [sperm and egg] and had a successful birth. (Participant B)

The women often recounted painful experiences of the stigma that they had to face because of their inability to have children:

We tried and failed, and I was upset every month. My husband said it didn’t matter without children. I couldn’t accept the lack of children in the family. He suggested adopting other children, and I refused. In my mind, adoption is not the same. Now I can finally go to parents with pride, instead of hiding like before. Previously when my families talked about children, I used to find excuses to run away because I was worried that they would pay attention to me. I even did not want to go home for the New Year celebration a few times because it was too upsetting, although I understood that families were just expressing their care. After so many stressful years, we now finally have our own children. (Participant K)

#### Return to a normal life track

The women also felt that a safe childbirth meant they could return to a normal life after infertility and the treatment that disturbed their work routine and family life. This was especially true for those who had to set aside their work and careers to pursue ART:

I applied for leave since getting pregnant to avoid premature delivery. After giving birth I wanted to look after the baby myself. I am currently on maternity leave with work being suspended and salary not paid. Once the maternity leave ends, I will go back to work and return to the life track. (Participant A)

I put my career on hold and prioritised pregnancy, because it was not easy to get pregnant, uterine fibroids were growing at that time, and with the consideration of my maternal age. Now that pregnancy and birth ended successfully, if possible, I would like to return to work…. I will want to return to the normal life track. (Participant F)

## Discussion

This study builds on previous work on the psychosocial impact of ART treatment by exploring the experiences of first-time mothers who successfully conceived using ART treatment in Taiwan during pregnancy and transition to motherhood. As this was a qualitative study based on the experiences of a sample of 12 participants, our findings may not necessarily be representative of the experiences of all women undergoing ART treatment in Taiwan or elsewhere. However, our methods created a suitable “space” for these women to freely express their experiences and in-depth perspectives. This study also provided the opportunity to explore first-time mothers’ perspectives both during pregnancy and in the early stages of parenthood.

The key themes reflected in the interviews on how the women viewed themselves differently from mothers who became pregnant naturally, how they ensured the health and safety of the foetus, and their experiences of welcoming the new lives with excitement provide useful insights for policy and practise in this area. Research has consistently shown the adverse effects of infertility on the emotional well-being of couples, especially women, as they experience feelings of disappointment, social isolation, distress, low self-esteem, and a sense of stigma [8, 18, 22]. Although our study did not directly explore the impact of infertility, the accounts clearly reflected how the subjects experienced these issues as a result of their infertility.

As indicated by other researchers [3], pregnancy carried a different meaning for the women in our study. After successfully becoming pregnant after long periods of infertility, they viewed themselves as different from women who conceived naturally. Although pregnancy made them feel excited and happy, they were gripped with a fear of losing the foetus, often battling with feelings of uncertainty about the pregnancy outcome.

The heightened sense of vulnerability these mothers experienced during pregnancy and their constant fears about pregnancy loss, preterm delivery, and the impact of ART on their own health and that of their babies could all have contributed to the increased levels of anxiety among women who conceived using ART compared to those who conceived naturally, as reported in previous studies [3, 4, 9, 17]. The constant psychological pressure that these women felt to have a successful pregnancy could contribute to their anxiety. The safety and health of the foetus was a primary concern for the women in our study. They resorted to all possible measures that they thought would ensure the health of the foetus. We did not find any indication of higher levels of distress during pregnancy compared to the previous state of being infertile as suggested by other researchers, however [8]. The impact of Taiwanese society on pregnancy following ART has been indicated by a previous study [17]. Among our participants, the pregnancy was viewed as a family event with intense practical and emotional support provided for the women from the extended family. This was also a major resource that helped the women cope with the demands of the ART treatment and pregnancy. The social impact on the pregnancy was also evident as some women viewed the birth of a child as an opportunity to prove their fertility to family and friends.

Following the birth of the baby, women in our study viewed the hardships as worthwhile and felt a strong sense of satisfaction embarking on the journey of motherhood. They tended to anchor their feelings similar to a ‘future of hope’ as reported by other researchers [16]. Although we did not directly explore parenting morale among the women, it would appear from the accounts that they had a strong parenting morale during their transition to motherhood [13]. While the arrival of the baby created a sense of a complete and cohesive family, the mothers did not express any concerns about the child’s gender and loss of freedom in their future lives as parents [9]. As our findings indicated, ART treatment could cause huge stresses on women’s psychosocial health, but the women in this study seemed to be remarkably resilient to parenting stress during their transition to motherhood [23].

This is one of the very few qualitative studies that have explored the experiences of women who conceived using ART in Taiwan. The qualitative approach offered the opportunity for an in-depth exploration of the experiences, but the findings may not be considered generalisable due to the qualitative nature of the study and the purposive sampling method adopted. The likely influence of the interviewer on the research process is another limitation that needs to be considered in qualitative research [24] as the relationship may encourage or inhibit the interviewee to share their personal and intimate experiences with the interviewer. The first author (MzH) who conducted the interviews worked as a consultant at the infertility unit from where the women were recruited. While this helped the interviewer to build a trusting relationship with the interviewees and facilitated the recruitment and the interviews, this might have introduced some courtesy bias into the findings. In order to enhance the reliability of the results, the researcher invited the participants to validate the accuracy of the preliminary findings. Regular team discussions including experts from different disciplines including qualitative methods, obstetric nursing, infertility, and maternity care also facilitated the quality of the overall research process.

## Conclusion

Overall, the key findings from our study demonstrated that the experience of pregnancy following ART involved mixed feelings and emotions during the various stages of pregnancy and during the participants’ transition to motherhood. The women’s perceptions of being different from mothers who became pregnant naturally, the intense anxieties they experienced as the pregnancy progressed, and their overriding excitement and satisfaction at the arrival of the baby are all useful indicators for policy and practise in this area. Our findings, in particular the intense anxieties that followed pregnancy and the efforts the mothers undertook to ensure the health and safety of their foetus, indicate the need for educational as well as other interventions to support these mothers physically and psychologically during the course of ART. On a broader level, the findings indicate the need to address the stigma related to infertility. The impact of the family and the support that the women received while pregnant and in their transition to parenthood is an aspect to consider and build on when planning psychosocial intervention programmes. Future qualitative studies should explore perspectives of fathers and the extended family members and employ longitudinal approaches to capture the long-term experiences of the women following ART treatment.

## Abbreviation

ART: Assisted reproductive technology
